# Estonia advances on SDG achievement by tackling high alcohol consumption through a multi-pronged approach

**DOI:** 10.1093/eurpub/ckaa031

**Published:** 2020-05-11

**Authors:** Triinu Täht, Kristina Köhler, Elen Ohov, Bettina Menne, Francesco Zambon, Leda Nemer

**Affiliations:** c1 Public Health Department, Ministry of Social Affairs, Tallinn, Estonia; c2 Analysis and Statistics Department, Ministry of Social Affairs, Tallinn, Estonia; c3 EU Affairs and International Cooperation Department, Ministry of Social Affairs, Tallinn, Estonia; c4 Sustainable Development and Health, Division of Policy and Governance for Health and Well-being, WHO Regional Office for Europe, Copenhagen, Denmark; c5 Investment for Health and Development in Healthy Settings, WHO European Office for Investment for Health and Development, Venice, Italy

## Abstract

Estonia has implemented a comprehensive, multipronged approach to the reduction of alcohol consumption in the population, comprising a series of successful policy responses. The Estonian alcohol strategy (2014) builds on the Global strategy to reduce the harmful use of alcohol and the European action plan to reduce the harmful use of alcohol 2012–2010. It aims to decrease the overall yearly consumption of alcohol among the adult population to less than 8 litres of absolute alcohol per capita. Gathering support across society from a range of stakeholders, including policy-makers, researchers, parents and advocates, has been one of the key elements in the implementation of the policy. High-level political commitment and strategic timing of efforts have maintained the issue of alcohol control on the political agenda and in the public’s mind.

## The issue

Alcohol is linked to more than 200 diseases and health conditions and contributes significantly to the global burden of non-communicable diseases. It also creates a number of social problems and hampers economic development. Estonia has suffered greatly from alcohol-related harm. Ten years ago, Estonia had one of the highest levels of alcohol consumption in the world with patterns characterized by heavy episodic drinking, often starting at a young age. Historically, alcohol consumption levels in Estonia were similar to those in countries of northern Europe and the former Soviet Union. According to the Health Behaviour in School-aged Children (HBSC) 2013/2014 study, 84.3% of 15-year olds, 60.8% of 13-year olds and 31% of 11-year olds had tried alcohol*.*^1^^,^^2^

By implementing a comprehensive evidence-based alcohol policy, Estonia has reduced alcohol consumption by one-third over the last decade. As a result, alcohol-related morbidity and mortality have decreased and the health gap between different population groups has narrowed. Estonia’s alcohol policy is directly linked to implementation of Sustainable Development Goal (SDG) 3 (good health and well-being), and specifically target 3.5, which aims to ‘strengthen the prevention and treatment of substance abuse, including narcotic drug abuse and harmful use of alcohol’. Since alcohol-related deaths and health problems are more pronounced at the lower end of the social gradient, reducing the harmful use of alcohol will also contribute to achieving SDG 10 to ‘reduce inequality within and among countries’.^3^

## The process

Estonia had no official alcohol policy until 2014. Earlier efforts to put stricter alcohol-control policies in place had been met with a lack of public support and little political interest. Taxing alcohol was the first measure to be taken and has the highest approval rates among politicians. Yet, until recently, tax hikes have not been able to keep up with income growth.

The process of developing the Estonian alcohol strategy^4^ focussed on gathering knowledge, creating expertise, raising public awareness and building social demand for stricter policy in this area. In line with the strategic directions of the WHO Roadmap to implement the 2030 Agenda for Sustainable Development,^5^ building on Health 2020, the European policy for health and well-being^6^ on advancing governance and leadership for health and well-being, dialogue among the stakeholders was a key feature of the development and implementation of the strategy. As it includes action in 10 policy areas across government (including health, education, economy and finance), it also promotes a multisectoral approach.

The Estonian alcohol strategy has four salient features:

1. Knowledge and expertise. The WHO Global strategy to reduce harmful use of alcohol^7^ and the 10 priority areas for evidence-based policy in the European action plan to reduce the harmful use of alcohol 2012–2020^8^ were the basis for the development of the Estonian alcohol strategy.^4^ During the process, the WHO Regional Office for Europe provided expertise and support in capacity building through ad-hoc consultations and the compilation of evidence to inform policy decisions.
*Source*: Public campaign by Road Safety Administration.

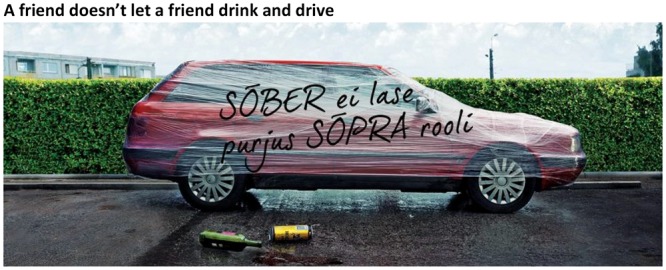

2. Involving stakeholders. A wide-ranging consultation process was carried out, involving all stakeholders. Working groups, comprising representatives of different ministries, government institutions, non-governmental organizations and the alcohol industry, were established for the strategy’s 10 action areas.
3. Governance. Implementation structures include the Ministerial Steering Group for Implementation of the Alcohol strategy, which reports to the Government. The Estonian alcohol strategy^4^ is also an integral part of the National Health Plan 2009–2020. A designated monitoring system measures the impact of the activities implemented. The Steering Group assembles regularly and presents annual progress reports to the Government. Alcohol consumption, marketing developments and health and alcohol-related social harm are also monitored annually.
4. Enhancing social demand and empowering the health sector. The roles of the general public and civil society have been instrumental in leading the public debate and raising awareness. In 2009, the first media campaign on alcohol resounded very strongly in society, immediately bringing alcohol-related harm into the spotlight. Since the economic sector was quick to react, measures could be taken promptly to provide an arena where stakeholders could discuss and support relevant health projects in civil society.

## Achievements to date

**Figure 1:**
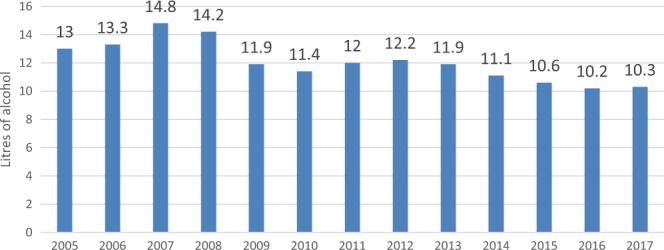
Alcohol-consumption trends per adult, Estonia, 2005–2017 *Source:* Alcohol market, consumption and harms in Estonia, Yearbook 2018.^9^

In the course of 10 years, the annual adult consumption of alcohol in Estonia dropped by almost a third (from 14.8 l in 2007 to 10.3 l pure alcohol per person in 2017) (figure 1).^9^ In the same period, mortality from alcohol-related diseases decreased by one-third and alcohol-related injuries and crime also decreased. In addition, the gap between life expectancy for men and women narrowed (from 11 to 9 years), the biggest contributor being changes in the levels of men’s alcohol consumption and smoking.^1^ While the reduction in overall consumption began as a sharp drop at the start of the economic crisis in 2008, its momentum was sustained through policy action.

## Conclusion

Estonia’s alcohol policy will continue to evolve. Several key lessons can be learnt from Estonia’s experience in developing and putting it in place

- High-level personal commitment raises the issue of alcohol control on the political agenda. The Minister of Health and Labour championed the topic of alcohol consumption at both the national and European Union (EU) levels in making it a priority of the Estonian Presidency of the Council of the European Union in 2017. This provided the support needed to put measures in place that will change alcohol consumption in Estonia.
- Involving the general public and civil society helps raise awareness about harmful alcohol consumption and strengthens public debate. In Estonia, open dialogue with stakeholders and the active participation of civil society were instrumental in garnering public support and changing people’s attitudes about the harmful use of alcohol. Optimal timing and building momentum at many levels are key to lowering alcohol consumption.
- Optimally timed, multifaceted, momentum-building efforts at different levels are effective in lowering alcohol consumption. In Estonia, alcohol consumption has declined by almost one-third since the 2008 financial crisis. While the economic situation triggered this reduction, its continuation can be attributed to: awareness raising efforts; education programmes for parents; rehabilitative training programmes for people charged with drink-driving; and the effects of an array of synergetic legislative changes to lower alcohol consumption.
- Consider the ‘side effects’ of alcohol-policy implementation. The tax measures implemented as part of the alcohol policy led to a two-fold difference between alcohol prices in Estonia and Latvia and an unintended increase in cross-border trade between the two countries. This resulted in public discussion about pricing policies and a decrease in public support of tax increases.

As a response to the increased cross-border alcohol purchases from Latvia, the new Government, formed after the March 2019 parliamentary elections, decided to reduce the excise duty on beer and spirits by 25% as of July 2019 with the aim of reverting. The aim has been to revert the cross-border trade and to regain the state budget revenues. In return, alcohol excise duties were reduced also in Latvia. The effects of this policy change on cross-border trade, on the overall consumption of alcohol and related health impacts remain to be seen. It is important to emphasize; however that the setbacks related to the tax policy measures, have not reverted the alcohol policy in its entirety, underpinning the importance of a comprehensive set of different policy measures.

## Disclaimer

The authors alone are responsible for the views expressed in this article, and they do not necessarily represent the views, decisions or policies of the institutions with which they are affiliated.

*Conflicts of interest*: None declared.
